# Virtual screening, identification and experimental testing of novel inhibitors of PBEF1/Visfatin/NMPRTase for glioma therapy

**DOI:** 10.1186/2043-9113-1-5

**Published:** 2011-01-20

**Authors:** Nagasuma Chandra, Raghu Bhagavat, Eshita Sharma, P Sreekanthreddy, Kumaravel Somasundaram

**Affiliations:** 1 Bioinformatics Centre, Indian Institute of Science, Bangalore 560012, India; 2 Microbiology and Cell Biology, Indian Institute of Science, Bangalore 560012, India

## Abstract

**Background:**

Pre-B-cell colony enhancing factor 1 gene (PBEF1) encodes nicotinamide phosphoribosyltransferase (NMPRTase), which catalyses the rate limiting step in the salvage pathway of NAD^+ ^metabolism in mammalian cells. PBEF1 transcript and protein levels have been shown to be elevated in glioblastoma and a chemical inhibitor of NMPRTase has been shown to specifically inhibit cancer cells.

**Methods:**

Virtual screening using docking was used to screen a library of more than 13,000 chemical compounds. A shortlisted set of compounds were tested for their inhibition activity *in vitro *by an NMPRTase enzyme assay. Further, the ability of the compounds to inhibit glioma cell proliferation was carried out.

**Results:**

Virtual screening resulted in short listing of 34 possible ligands, of which six were tested experimentally, using the NMPRTase enzyme inhibition assay and further with the glioma cell viability assays. Of these, two compounds were found to be significantly efficacious in inhibiting the conversion of nicotinamide to NAD^+^, and out of which, one compound, 3-amino-2-benzyl-7-nitro-4-(2-quinolyl-)-1,2-dihydroisoquinolin-1-one, was found to inhibit the growth of a PBEF1 over expressing glioma derived cell line U87 as well.

**Conclusions:**

Thus, a novel inhibitor has been identified through a structure based drug discovery approach and is further supported by experimental evidence.

## Background

Gliomas are primary malignant tumors, originating in the brain, and account for 80% of adult primary brain tumors. The prognosis for patients with glioblastoma multiforme, a virulent variety of the disease is rather poor, with a median survival of less than one year [1]. Several molecular and biochemical abnormalities such as specific chromosomal aberrations, upregulation of epidermal growth factor receptor (EGFR), loss of phosphate and tensin homology (PTEN), have been clearly associated with gliomas. Some pathways associated with higher grade gliomas are upregulation of PDGFRA (platelet derived growth factor receptor α), CDK4 (cyclin dependent kinase 4) and down-regulation of retinoblastoma (RB1) [1]. NAD^+ ^biosynthesis has been shown to be activated in cancers [2]. NAD^+^, in addition to its role as a redox cofactor, is also used as a substrate in several biochemical reactions including mono- and poly-ADP ribosylation (ART and PARP catalyzed), protein deacetylation and ADP-ribose cyclization [3]. NMPRTase catalyzes the conversion of free nicotinamide to nicotinamide mononucleotide (NMN), which is a key step in the salvage pathway of NAD^+^. Expression levels of NMPRTase (also known as visfatin/Pre B-cell enhancing factor1 (PBEF1)), was found to be upregulated in colorectal cancers [4], suggesting that NMPRTase may be crucial for maintaining cellular NAD^+ ^levels in tumors. Microarray analyses of glioma cells (grade II to IV) versus normal brain glial cells has identified differential expression of NMPRTase in glioma with 2-5 fold upregulation in glioma cells, depending on the grade of the tumor (increased expression of NMPRTase with greater progression of the disease, Grade IV > Grade III > Grade II) [5]. Observation of increased rates of NAD^+ ^metabolism in glioma, suggests that the cancer cells may be critically dependent upon metabolites produced in the pathway, and presents a possible strategy to counter the disease, through the inhibition of key enzymes in the pathway.

The crystal structures of free NMPRTase, NMPRTase bound to NMN, and NMPRTase bound to the inhibitor FK866 [6], have been recently reported. FK866 is a potent small-molecule inhibitor of human NMPRTase, and the consequent reduction in NAD^+ ^levels can cause apoptosis of tumor cells while having minimal effect on normal cells [7]. FK866 also turns out to be the only promising inhibitor known, for the enzyme. The structures provide a basis for understanding substrate specificity, mechanism of enzyme action, and hence provide a framework for design of novel NMPRTase inhibitors.

An extremely useful step in the rational design of inhibitors is to utilize the three dimensional structural information of the target protein and identify a possible lead compound from large libraries of compounds. The most efficient method to do this, of course would be to carry out virtual screening. In the recent years several docking algorithms have been developed which are being used for virtual screening of potential ligands to a given protein at the three dimensional level [8,9]. We recently developed a parallel version of a popular docking algorithm - AutoDock [10] and have implemented this on an IBM Bluegene supercomputer [11], rendering the docking approach amenable for high throughput virtual screening. Here we report virtual screening of a large library of compounds and short listing of six candidate molecules that are likely to bind to NMPRTase. These compounds were subsequently tested experimentally for their ability to (a) inhibit the conversion of nicotinamide to NAD^+ ^by NMPRTase and (b) inhibit efficiently the growth of a PBEF1 over expressing glioblastoma derived cell line U87. Based on these studies, a promising lead compound has been identified.

## Methods

### Reagents and cell lines

Cancer cell lines U373, U138, LN229, U343, U87, U251 and LN18 (all glioma derived cell lines), SW480 (colon carcinoma), HaCat (immortalized human keratinocytes) and HBL100 (immortalized human breast epithelial cells) cells were cultured in Dulbecco's Modified Eagle Medium (DMEM) respectively with 10% Fetal bovine serum, penicillin and streptomycin at 37 °C in a humidified atmosphere with 5% CO_2_. The six best docked compounds were purchased from Maybridge, Thermo Fisher Scientific, UK. C14-Nicotinamide (Specific activity 55 mCi/mmol) was purchased from American Radio labeled Chemicals, USA.

### Virtual screening

Different steps involved in virtual screening are briefly described below:

#### a. Selection of ligand library and preparation of ligands and protein

Virtual screening was performed to identify possible lead compounds from the Maybridge *HitFinder*™ database. The Maybridge *HitFinder*™ sets are structural representatives of large non-redundant chemical libraries. This collection includes 14,400 compounds that represent the drug-like diversity of the Maybridge Screening Collection (~56,000 compounds). All the screening compounds fit Lipinski guidelines for drug-likeness; partition coefficient, ClogP ≤ 5, H-bond donors ≤ 5, H-bond acceptors ≤ 10, molecular weight ≤ 500 [12]. The Maybridge *HitFinder*™ set was obtained from http://www.maybridge.com. The ligand files were prepared for docking using Schrodinger *Ligprep *software [13]. In addition to the generation of energy minimized 3D structures, Schrodinger *Ligprep *was also used for addition of hydrogens and desalting of metal ions. The main objective of using LigPrep was to obtain low energy 3D structures of the set of ligands in the library, for use in further computational studies. OPLS_2005 force field was utilized to optimize the geometry and minimize the energy. Force field parameters were assigned to the ligand atoms using default treatment for possible tautomers, and ionization at a selected pH range (7 ± 2 by default), and ring conformations (1 ring conformer by default).The *Ligparse *module was used during Ligprep and the ligands with following properties were removed from the set: molecular weight less than 200, number of neutral acceptor groups greater than 10 and number of neutral donor groups greater than 5. A total of 13214 ligands were selected and retained out of the total 14,400 original ligands.

#### b. Docking

The co-ordinates of the human NMPRTase (2GVG- complex with the reaction product nicotinamide mononucleotide; 2GVJ- complex with an inhibitor, n-[4-(1-benzoylpiperidin-4-yl)butyl]-3-pyridin-3-ylpropanamide- FK866 were obtained from Protein Data Bank (PDB) [14]. The protein file was prepared for docking by removal of water molecules, addition of polar hydrogens, removal of ligand and phosphate groups in active site, and addition of Kollman charges [15]. The macromolecule was treated to be completely rigid for all docking studies to reduce the extensive computational costs. A grid box encompassing both the NMN and FK866 sites (86×60×50; 0.375 Å spacing) was constructed and used for all the docking runs. Our definition of the site as input for the docking program encompasses the phosphate site completely, since the grid box with dimensions 80 × 60 × 50 points with a default spacing of 0.375 Å, is sufficiently large to encompass the entire binding pocket and any nearby minor sites such as that of the phosphate site. Thus, the search space for generating ligand map files using Autogrid, is big enough to encompass the PO4 site, and is not neglected. Docking parameter files were prepared for each ligand using the following parameters: ga_pop_size 150; ga_num_evals 2500000; ga_num_generations 500; ga_run 100 and rmstol 1.0. The Maybridge *HitFinder*™ dataset was docked using the parallel version of AutoDock 3, available in the laboratory using 256 processors on an IBM cluster. This process greatly reduced the computational cost and time involved in virtual screening of the large dataset (~13214). Clustering was performed based on the similarity in binding modes and affinities in the run cycles. The size of the clusters refer to the total number of conformations of the ligand that bind in the same orientation within the specified RMSD threshold (1 Å was used in our study) and binding with the same energy. Thus, a cluster is defined as a unit of such similar confirmations. More the number in each cluster better is the accuracy and confidence of the predicted pose of the ligand molecule. The *Ligand Protein Contacts *(LPC) [16] was used for obtaining the interactions of docked ligand atoms with the macromolecule, hydrogen bonding, van der Waals contacts and the solvent accessible surface area.

#### c. Short listing of potential leads

The docking log files (.dlg) were parsed using in-house perl scripts to scan the clustering histograms, and identify ligands that have docked poses with binding energy lower than the cut-off criteria and cluster size greater than the defined cut-off (See Table 1). The cut-off values were obtained from docking the known inhibitor FK866, and product NMN, to the receptor, and retrieving the docking energy and cluster size values for poses that have least deviation from the crystal pose (RMSD < 1.0). As a newer version of AutoDock became available during the course of this work, the docking exercise was repeated for the short listed compounds with the same parameters using AutoDock4 (version 4.0.1).

**Table 1:** Docking results of control compounds

				
	**Biochemical energy of binding (kcal/mol)**	**AD4 docked (kcal/mol)**	**RMSD from crystal pose**	**Size of cluster**

NMN	-	-8.02	0.87	35

FK866	-9.25	-8.63	0.77	16

#### Energy minimization

Minimization of the docked ligands in the best ranked poses was done using CNS software suite [17]. Conjugate gradient method was used for minimization with flexibility allowed only for those atoms within the 6 Å radius of every atom of the ligand for 150 runs. The topology and parameter files for the compounds were obtained from XPLO-2D software [18]. Molecular visualization tool Pymol [19] was used to generate the images of the docked complexes.

### RNA isolation and RT-qPCR

RNA isolation and RT-qPCR were carried out as described before [5]. Total RNA was extracted from cancer cell lines by using the TRI reagent (Sigma). The RNA samples were quantified by measuring the absorbance using a spectrophotometer and visualized on a MOPS formaldehyde gel for quality assurance. The relative quantification of the expression levels of selected genes was carried out using a two-step strategy: In the first step, cDNA was generated from RNA derived from different tissue samples using a cDNA archive kit (ABI PRISM); subsequently, real-time quantitative PCR was carried out in an ABI PRISM 7900 (Applied Biosystems) sequence detection system with the cDNA as template using PBEF1 specific primer set and a Dynamo kit containing SYBR green dye (Finnzymes). All measurements were made in triplicates. The genes GARS (glycyl-tRNA synthetase), AGPAT1 (1-acylglycerol-3-phosphate O-acyltransferase 1), ATP5G1 [ATP synthase, H^+ ^transporting, mitochondrial F0 complex, subunit C1 (subunit 9)], and RPL35A (ribosomal protein L35a) were used as internal controls because their expression levels were found to be unaltered in microarray experiments. The fold change (log2 ratio) in PBEF1 gene expression was calculated over its mean expression in normal brain samples obtained from previously published results [5]. Delta-delta CT method was used for the calculation of ratios. Sequences of reverse transcription-PCR primers and conditions used will be provided on request.

### Western blot analysis

Western blot analysis was performed as described previously [5] with rabbit polyclonal antibody against GST-PBEF1 raised in the laboratory using a standard immunization protocol and antitubulin antibody.

### NMPRTase assay

The measurement of NMPRTase activity was carried out as described before [7]. To prepare cytoplasmic extract, as source of NMPRTase, we collected logarithmically growing U87 glioblastoma cells by centrifugation and washed three times with Ca^2+ ^Mg^2+ ^free PBS. The cells pellet (2-3 × 10^7 ^cells) was suspended and lysed in 1 mL of 0.01 M NaH_2_PO_4 _(pH 7.4) by one round of freezing and slow thawing. The clear supernatant was recovered on ice after centrifugation at 23,000 × g at 0°C for 90 minutes. 70 mL of 1% protamine sulfate was added per ml of supernatant and incubated for 15 min on ice, followed by centrifugation at 23,000 × g at 0°C for 30 minutes. The final supernatant was stored in small aliquots at -80°C. The NMPRTase activity was determined in 0.5 mL of reaction solution consisting of 5 mM MgCl_2_, 2 mM ATP, 0.5 mM phosphoribosyl PPI, 0.1 mM 14[C]-nicotinamide (specific activity: 50 mCi/mmol; American Radio labeled Chemicals, Inc.) and 50 mM Tris (pH 8.8) at 37°C. The reaction was started by adding 100 μL of cell extract and stopped after 1 hr with excess of cold nicotinamide and heating (2 min, 105°C). The precipitate was removed by centrifugation at 2500 × g at 4°C for 10 min and the supernatant was stored at -20°C. The 14[C]-labeled components in the cell extracts were separated and identified using thin-layer chromatography (cellulose/1 M ammonium sulphate:ethanol (3:7)). The chromatograms were run, exposed to imaging plates (Fuji) and read using Phosphorimager (Fuji). The 14[C]-labeled NAD^+ ^was quantified using Alpha Innotec software.

### MTT assay

MTT assay was carried out as described previously [20]. A total of 1.5 × 10^3 ^cells/well were plated in a 96-well plate. After 24 h of plating, the cells were treated with indicated amounts of compounds. A measure of 20 μL (5 mg/mL) of MTT was added to each well 48 hrs after the addition of the compounds. MTT is a tetrazolium salt that is converted by living cells into purple formazan crystals. The medium was removed from the wells 3 hrs after MTT addition and 200 μL of DMSO was added to dissolve the formazan crystals, and then the absorbance was measured at 550 nm in an ELISA reader.

## Results

### Description of the binding site

The crystal structures of NMPRTase, in complex with the known inhibitor FK866, and reaction product NMN, reveal that the active enzyme exists as a dimer. The structures of the two complexes were very similar to each other and no significant conformational changes were observed upon ligand binding [6], making it meaningful to compare the binding modes of the two ligands. The catalytic centre is present at the interface of the two chains. There are two active sites per dimer and residues from both the chains are involved in the interactions with the product NMN at each site [6]. The binding pockets of the two ligands overlap partly with each other, together extending into a single large pocket resembling a long tunnel, with NMN binding at one end and FK866 spanning till the other end. Some residues are common to both, indicating the overlap in their binding poses. An essential feature of both the binding poses is the presence of hydrophobic stacking in which an aromatic group in the ligand is sandwiched between F193 of one subunit and Y18 of another subunit. The conservation of these interactions, especially the hydrophobic stacking, was used as a criterion for filtering docked ligands, subsequent to the selection on the basis of binding energy and cluster size. The uniqueness of the binding site has been studied by comparing NMPRTase with closely related NAPRT and QPRT enzymes, present in the same biochemical pathway [6]. A multiple sequence alignment of the human NMPRTase with the other two proteins showed that they have diverged in terms of sequences considerably, but adopt the same fold. Yet, analysis of their binding sites using PocketMatch [21], indicates that considerable difference exists at the binding site level, suggesting that design of specific inhibitors can be achieved. It has also been reported earlier that FK866, a potent inhibitor of NMPRTase does not inhibit NAPRT, consistent with this observation [6].

### Identification and analysis of potential compounds

As a control study, the enzyme's reaction product NMN as well as the known inhibitor, FK866 were docked to the protein, an exercise which resulted in reproducing the crystal structure poses for both compounds. Table 1 lists the interaction energies computed for the docked NMN and FK866 as well as the deviations from the crystallographic observed poses. The energy values computed for these reference compounds were used as reference values for identifying possible ligands from the large compound library. All those compounds which exhibited interaction energies above this threshold or in other words indicated binding weaker than the reference compounds were eliminated from the list for further analysis. The result of the virtual screening of the dataset is summarized in Table 2.

**Table 2:** Virtual Screening results

	#ligands	Energy cut-off	# ligands above cut-off	Cluster size cut-off	Potential ligands
*Maybridge HitFinder*™	13214	< -8.5 kcal/mol	34	> 15	6

Summary of docking results; (1) Docking of reference compounds to NMPRTase reproducing the crystal poses; (2) An overview of results of virtual screening to the same protein molecule.

**Figure 1:**
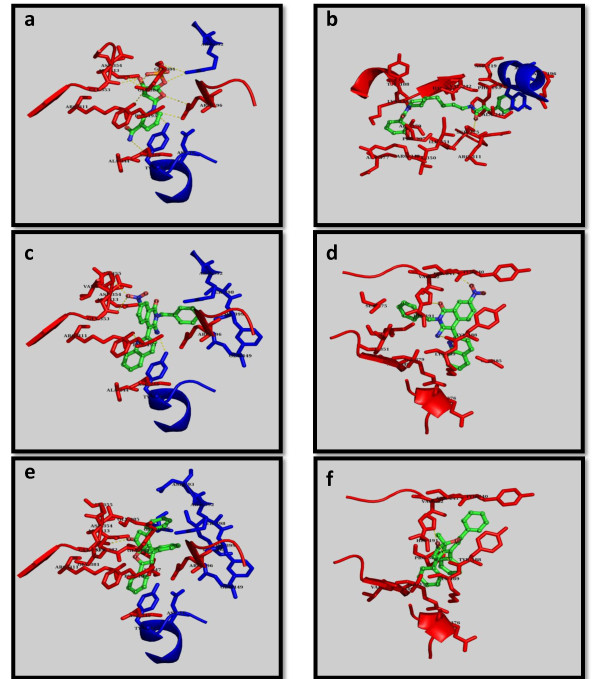
**Crystal poses of the Control compounds and docked poses of the identified compounds**. Crystallographically observed binding modes of the known and new ligands in NMPRTase; (a) NMN, (b) FK866, and docked binding modes of compounds 4 and 5. (c) and (e) panels indicate the first binding modes of compounds 5 and 4 respectively while panels (d) and (f) indicate the second binding modes of compounds 5 and 4 respectively. The ligands are in ball and stick representation and colored by standard atom types; the A chain residues of the site are shown in red and C chain residues are in blue, in all the panels.

**Figure 2:**
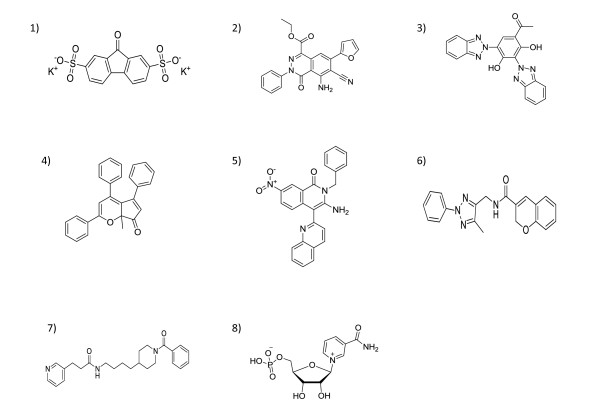
**2D- structures of compounds**. 2D-structures of the six compounds along with the control compounds FK866 and NMN; panels numbered 1 to 6 indicate the 2D-structures for compounds 1-6, and panels numbered 7 and 8- for FK866 and NMN respectively.

For selection of potential ligands, analysis of ligand protein contacts for top ranking poses of every ligand was carried out and the interactions of docked compounds were visualized. Interactions conserved with NMN and FK866 binding were calculated and compared with that of the short listed compounds. The best poses were identified using the following criteria in the given order of preference i) lowest binding energy in the largest sized cluster ii) number of hydrogen bonds with the active site residues and iii) conservation of interactions with those from NMN/FK866 binding. Preference is given to the largest sized least binding energy cluster and then examined to verify if one or more hydrogen bonds are conserved between the natural substrate NMN, or the previously known inhibitor FK866. This was to ensure that the ligands shortlisted were actually docking into the binding site of interest. All the 34 shortlisted from an initial list of 13214 compounds pass these criteria and is shown in Table 2. The top six in this list that were readily purchasable were considered for further studies which are shown in Table 3. The docking results for all the six compounds showed that docked poses passing the defined thresholds were found to cluster into two main groups, corresponding to the binding modes of NMN and FK866 respectively, with docking energies nearly comparable between the modes for each ligand. The binding sites for both possible modes are shown in Figure 1 and Additional file 1, Figure S1, whereas the list of interactions at the site for both possible modes are indicated in Table 3, and illustrated in Figure 1. 2D-structures of the six compounds and the two control compounds, FK866 and NMN are shown in Figure 2.

**Table 3:** Binding Free Energy for the six identified compounds and the control compounds

Comp no.	Compound name	Binding Free energy (kcal/mol)	Number in clusters	Residues in the binding pockets
1	Dipotassium 9-oxo-9H-fluorene-2,7-disulfonate	-8.56 (Mode I)	54	**F**_**193**_, **R**_**196**_, G_197_, **R**_**311**_, **G**_**353**_, V_356_**, G**_**384**__, _**Y**_**18**_^**1**^, T_391_^1^, **R**_**392**_^**1**^, **D**_**393**_^**1**^
		
		-7.98 (Mode II)	32	Y_188_, H_191_, F_193_, D_219_, V_242_, A_244_, **A**_**245**_, S_275_, I_309_, **R**_**31**__1_, I_351_, Y_18_^1^

2	Ethyl-5-amino-6-cyano-7-(2-furyl)-4-oxo-3-phenyl-3,4-dihydro-1-phthalazinecarboxylate	-9.45 (Mode I)	44	**F**_**193**_, **R**_**196**_, D_219_, E_246_, **H**_**247**_, R_311_, **D**_**313**_, G_353_, **D**_**354**_, G_355_, V_356_, G_381_, S_382_, G_383_, G_384_, G_385, _D_16_^1^, Y_18_^1^, E_149_^1^, **K**_**415**_^**1**^, **K**_**423**_^**1**^
		
		-8.12 (Mode II)	14	Y_188_, **H**_**191**_, G_217_, Y_240_, S_241_, V_242_, P_273_, P_307_, I_309_, **R**_**34**__9_, V_350_, I_351_, I_378_, A_379_, Q_92_^1^

3	1,[3,5-Di(2H-1,2,3-benzotriazol-2-yl)-2,4-dihydroxyphenyl]ethan-1-one	-9.55 (Mode I)	18	**F**_**193**_, R_196_, **R**_**311**_, **G**_**353**_, D_354_, G_355_, V_356_, G_381_, S_382_, **G**_**383**_, G_385, _D_16_^1^, Y_18_^1^, R_40_^1^, **R**_**392**_^**1**^, D_393_^1^, N_396_^1^
		
		-8.69 (Mode II)	15	Y_188_, K_189_, **H**_**191**_, F_193_, G_217_, **D**_**219**_, Y_240_, S_241_, V_242_, A_244_, S_275_, I_309_, R_311_, R_349_, V_350_, I_351_, E_376_, N_377_, I_378_, A_379_, Y_18_^1^

4	7a-methyl-2,4,5-triphenyl-7,7a-dihydrocyclopenta[b]pyran-7-one	-8.56 (Mode I)	40	F_193_, **R**_**196**_, H_247_, **G**_**35**__3_, D_354_, G_355_, V_356_, G_381_, S_382_, G_383_, G_384_, G_385, _Y_18_^1^, R_40_^1^, **R**_**392**_^**1**^, S_398_^1^, K_415_^1^, K_423_^1^
		
		-8.23 (Mode II)	27	**Y**_**188**_, K_189_, **H**_**191**_, Y_240_, S_241_, V_242_, P_307_, I_309_, R_349_, V_350_, E_376_, I_378_, A_379_

5	3-amino-2-benzyl-7-nitro-4-(2-quinolyl)-1,2-dihydroisoquinolin-1-one	-10.54 (Mode I)	38	**F**_**193**_, **R**_**196**_, D_219_, A_244_, **H**_**247**_, **R**_**311**_, **D**_**313**_, **G**_**353**_, D_354_, **G**_**355**_, V_356, _**Y**_**18**_^**1**^, E_149_^1^, R_392_^1^, **S**_**398**_^**1**^, F_399_^1^, **K**_**415**_^**1**^, K_423_^1^
		
		-9.84 (Mode II)	16	G_185_, **Y**_**188**_, K_189_, **H**_**19**__1_, Y_240_, **S**_**24**__1_, **V**_**242**_, S_275_, I_309_, R_349_, I_351_, E_376_, A_379_

6	N-[(5-methyl-2-phenyl-2H-1,2,3-triazol-4-yl)methyl]-2H-chromene-3-carboxamide	-9.86 (Mode I)	16	F_193_, **R**_**196**_, **D**_**219**_, V_242_, A_244_, S_275_, G_353_, D_354_, G_355_, V_356_, G_381_, S_382_, G_383_, **G**_**384**_, G_385, _D_16_^1^, Y_18_^1^
		
		-9.64 (Mode II)	10	Y_188_, **H**_**191**_, F_193_, D_219_, **Y**_**240**_, S_241_, V_242_, A_244_, **S**_**275**_, I_309_, I_351_, Y_18_^1^, H_90_^1^, Q_92_^1^

7	**CONTROL**	-8.63	16	**F**_**193**_, G_194_, Y_195_, **R**_**19**__6_, G_197_, D_219_, **R**_**311**_, D_313_, G_353, _V_356_, G_384_, **G**_**385**_
	n-[4-(1-benzoylpiperidin-4-yl)butyl]-3-pyridin-3-ylpropanamide (FK866)			D_16_^1^, Y_18_^1^, **R**_**392**_^**1**^, D_393_^1^

8	**CONTROL**	-8.02	27	**F**_**193**_, **R**_**196**_, **G**_**353**_, D_354_, G_355_, **V**_**356**_, **D**_**357**_, **G**_**381**_, S_382_, G_383_, **G**_**384**_, **G**_**385**_, **K**_**38**__9_, **R**_**392**_^**1**^, D_393_^1^
	Nicotinamide ribose monophosphate (NMN)			

Binding energy for six compounds and control compounds NMN, and FK866 along with residues in each site. The subscript refers to the residue number. Residues involved in hydrogen bond interactions are shown in boldface.

### Inhibition of NMPRTase activity by selected *lead *compounds

**Figure 3:**
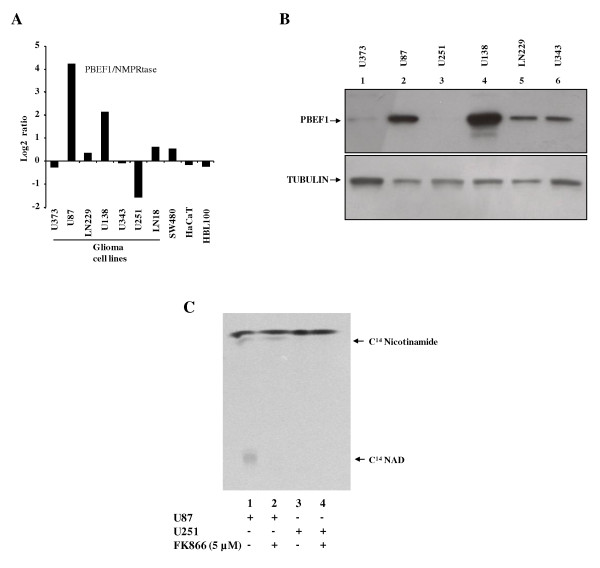
**NMPRTase transcript and protein levels in glioma cells and NMPRTase enzyme assay**. A. Log2-transformed gene expression ratios obtained from real-time quantitative PCR analysis are plotted for PBEF1. Each bar represents a data derived from the indicated cell line. In each sample, fold change in gene expression is calculated over its mean expression in normal brain samples. B. Equal amounts of total protein lysates from indicated cell lines were subjected to western blotting to detect levels of PBEF1 and Tubulin proteins. C. NMPRTase assay was carried out as described in the methods section with extracts obtained from U87 or U251 cells either with or without the FK866.

We then tested whether the selected lead compounds could inhibit NMPRTase activity. We measured the ability of NMPRTase to convert 14[C]-nicotinamide to 14[C]-NAD^+^. We had earlier shown that most of the glioblastoma tissues have elevated levels of transcript and protein of PBEF1/NMPRTase [5]. To prepare NMPRTase enzyme, we first tested a panel of glioma derived cell lines for NMPRTase transcript and protein levels. We found that, out of seven glioma cell lines tested, two cell lines, U87 and U138, had substantially high levels of PBEF1 transcripts in comparison to normal brain samples (Figure 3A). Western blotting analysis also corroborated above results that U87 and U138 had relatively higher levels of PBEF1/NMPRTase protein levels (Figure 3B).

**Figure 4:**
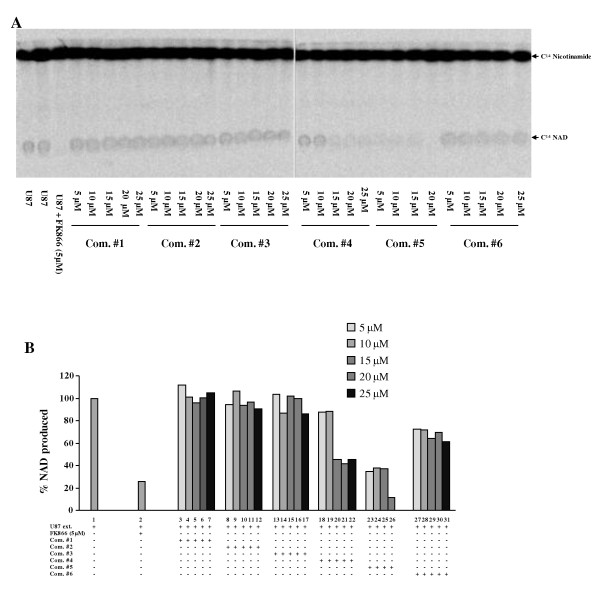
**NMPRTase inhibitor screen**. A. NMPRTase assay was carried out as described in the methods section with extracts obtained from U87 with indicated amounts of either FK866 or compound 1 to 6. B. The amount of C^14^NAD^+ ^formed in the experiment described above (A) is measured and shown. Please note that compound 5 and 4 inhibited the NMPRTase activity.

We chose U87 cells as the source of NMPRTase. The NMPRTase enzyme extract from U87 converted the 14[C] labeled nicotinamide to NAD^+ ^(Figure 3C lane 1). FK866, the known inhibitor of NMPRTase inhibited this reaction efficiently (Figure 3C compare lane 2 with 1). As expected, the extract from U251 cells, which had very low levels of PBEF1 transcript and protein, did not convert 14[C] labeled nicotinamide to NAD^+ ^(Figure 3C lane 3). We then tested the ability of six selected lead compounds to inhibit NMPRTase activity. We found that, of the six compounds tested, compounds 4 and 5 inhibited NMPRTase activity (Figure 4A and 4B) significantly. Compound 5 was found to be more potent in NMPRTase inhibition (Figure 4A and 4B compare lanes 23-26 with lanes 1 and 2).

### Inhibition of growth PBEF1 over expressing glioblastoma cell line U87

**Figure 5:**
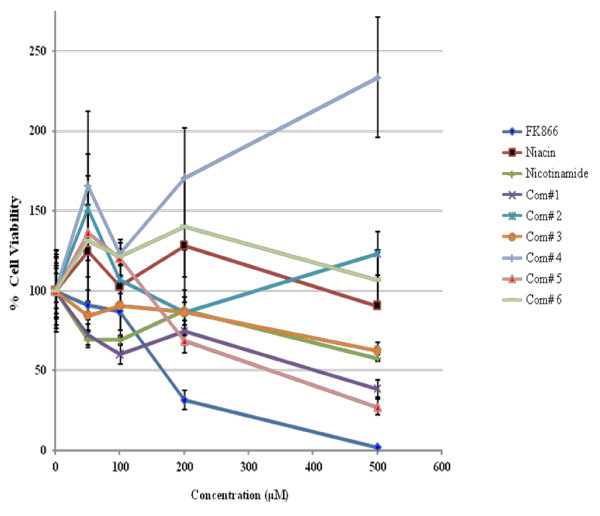
**Glioma cell growth inhibitor screen**. Viability was measured by MTT assay at 48 hrs after addition indicated compounds to U87 cells. The assays were carried out in triplicates and the mean value for each time point was used to generate the graph.

To correlate the NMPRTase inhibition property with cell growth inhibition, we then tested the ability of these compounds to inhibit the growth of a glioma derived cell line U87, which has elevated levels of PBEF1. FK866, the known NMPRTase inhibitor, inhibited the growth of U87 cells efficiently with an IC_50 _of 170 μM (Figure 5 and Table 4). Of the six selected lead compounds, we found that only compounds 1 and 5 inhibited the growth of U87 cells with an IC_50 _of 335 and 325 μM respectively (Figure 5 and Table 4). Since compound 1 did not inhibit NMPRTase activity (Figure 4), it might utilize a different mechanism to inhibit the growth of U87 cells. However, compound 5 inhibited NMPRTase activity as well as the growth of U87 cells. Further to confirm that inhibition of U87 cell growth by FK866 and compound 5 is because of their ability to inhibit NMPRTase, we tested the effect of these two compounds on the growth U251 cells which does not express NMPRTase. As expected, neither FK866 nor compound 5 inhibited U251 cells, while adriamycin inhibited very efficiently (data not shown). We thus conclude that compound 5 is a potent inhibitor of NMPRTase and cancer cell growth.

**Table 4:** IC_50 _values for the compounds

Compound Name	**IC**_**50 **_**(μM)**
FK866	170

Niacin	> 500

Nicotinamide	> 500

Compound 1	335

Compound 2	> 500

Compound 3	> 500

Compound 4	> 500

Compound 5	325

Compound 6	> 500

List of compounds and their IC_50 _values

## Discussion

Virtual screening procedure carried out resulted in short-listing of six compounds which were obtained and tested experimentally using both the enzyme inhibition assay as well as the cell growth inhibition assay. It is indeed gratifying that two of the six compounds clearly exhibit enzyme inhibition. This clearly demonstrates the usefulness of the docking based virtual screening approach used here, utilizing three dimensional structural information at atomic level detail.

Analysis of the docked poses indicate that two binding modes are observed within the large tunnel like binding pocket encompassing sites for both the natural product NMN and the partly overlapping larger inhibitor FK866. Given that the docking algorithm uses heuristics, a typical docking simulation is carried out by repeating each run about 150 times, each time using a different random number as a seed. This clustering pattern obtained subsequently for each docking simulation reflects the propensity of the ligand to occupy the binding site in the respective modes. Figure S1a-S1f shows the binding sites of all the six compounds. For all the six compounds tested, distinct clusters were observed indicating the two modes of binding as top ranking poses, the first overlapping in position with that of the natural product NMN and the second overlapping substantially with that of FK866. Figure S1i shows the superposition of the site of FK866 (as in 2GVJ) and the site corresponding to the second binding mode of compound 5, which clearly illustrates that the identified compound 5 occupies the same pocket as that of FK866.

The interactions observed in the first mode include a stacking interaction of the aromatic ring in the ligand with the side chains of F193 of one subunit and Y18 of another subunit. In the second mode, interactions with Y188, K189, Y240, S241, V242, S275, I309, R349, I351, and E376 are commonly seen. Figure 1 shows the binding sites of both modes for compound 4, 5; single modes of NMN (as in 2GVG) and FK866 (as in 2GVJ). It is interesting to note that majority of these residues are conserved in the NMPRTase proteins from several sources and their close sequence homologues.

Interestingly, Compound 5 docked with the lowest binding energy of all the compounds in both modes. It forms more number of hydrogen bonds with the binding site residues, all of them being crucial for NMN/FK866 binding. The two modes reflect the most plausible modes of binding for the compounds studied, and any one of them may be more important than the other for achieving inhibition. Irrespective of that, compound 5 is seen to be a better ligand in both the poses, further corroborated by the experimental studies. To consider the situation of the concentration of NMN being high in cellular environment, and hence the possibility of high population of NMPRTase molecules bound to NMN and subsequently any influence this might have for binding compound 5, we repeated the docking exercise with NMPRTase bound to product NMN and next with natural substrate nicotinamide. The second mode of binding for compound 5 was reproduced in this exercise, indicating that this compound will be capable of binding to the enzyme in the presence of NMN/nicotinamide also and will not have to compete with NMN/nicotinamide. This observation leads to favoring the second mode binding as being more important for achieving inhibition. Competition assays using enzyme kinetics has been carried out for FK866, but the interpretation of whether it is a competitive or non-competitive inhibitor is not clear [4,5]. It may also be possible that the compounds could inhibit the enzyme by both modes of action, thus exhibiting a 'mixed inhibition'. Despite these, the fact remains that both FK866 and the new compound identified in this study are effective in inhibiting glioma cell growth and this has been demonstrated to be mediated via inhibition of NMPRTase.

The compound 5 also exhibited highest efficacy in enzyme inhibition and in cell growth assay among all the compounds tested. This compound had, in addition to the hydrophobic and aromatic interactions, hydrogen bonding interactions with F193, G353, G384, R196, H247 and R311 of one subunit and Y18 of another subunit in the first mode; and Y188 and H191 in the second mode. The other compounds, though bind at the same location do not have hydrogen bonding moieties in them, perhaps explaining higher strength of binding and hence inhibition by compound 5.

## Conclusion

Virtual screening by molecular docking, carried out using AutoDock, has thus proved to be useful in short listing potential lead compounds. Recently Colombano and coworkers [22] reported the synthesis of FK866 analogues and showed that two of them were indeed good inhibitors of NMPRTase. All these are however based on the same scaffold. It is indeed useful to see that two new molecules belonging to different scaffolds have now been identified and tested through experimental methods. Compound 5 in particular, serves as a promising lead compound for furthering structure based discovery of drugs for use in glioma.

## List of Abbreviations

PBEF1: Pre-B-cell colony enhancing factor; NMPRTase: Nicotinamide phosphoribosyltransferase; GBM: Glioblastoma; ART: ADP-ribosyltransferases; PARP: Poly ADP Ribose Polymerase; MTT: 3-(4,5-Dimethylthiazol-2-yl)-2,5-Diphenyltetrazolium Bromide.

## Competing interests

The authors declare that they have no competing interests.

## Authors' contributions

RB and ES carried out docking and related studies; PS carried out glioma enzyme and cell based assays. NC and KS conceived of the study, and participated in its design and coordination and helped to draft the manuscript. All authors read and approved the final manuscript.

## Supplementary Material

Additional file 1 **Figure S1 Binding site of the six identified compounds and the two control compounds**. Figure showing the binding sites of all the top six compounds along with the control compounds, NMN and FK866. Click here for file

## References

[B1] Furnari FB Fenton T Bachoo RM Mukasa A Stommel JM Stegh A Hahn WC Ligon KL Louis DN Brennan C Malignant astrocytic glioma: genetics, biology, and paths to treatment Genes Dev 2007 21 21 2683 2710 10.1101/gad.1596707 17974913

[B2] Yang H Yang T Baur JA Perez E Matsui T Carmona JJ Lamming DW Souza-Pinto NC Bohr VA Rosenzweig A Nutrient-sensitive mitochondrial NAD+ levels dictate cell survival Cell 2007 130 6 1095 1107 10.1016/j.cell.2007.07.035 17889652 PMC3366687

[B3] Magni G Amici A Emanuelli M Orsomando G Raffaelli N Ruggieri S Enzymology of NAD+ homeostasis in man Cellular and Molecular Life Sciences 2004 61 1 19 34 10.1007/s00018-003-3161-1 14704851 PMC11138864

[B4] Jia SH Li Y Parodo J Kapus A Fan L Rotstein OD Marshall JC Pre-B cell colony-enhancing factor inhibits neutrophil apoptosis in experimental inflammation and clinical sepsis J Clin Invest 2004 113 9 1318 1327 15124023 10.1172/JCI19930 PMC398427

[B5] Reddy PS Umesh S Thota B Tandon A Pandey P Hegde AS Balasubramaniam A Chandramouli BA Santosh V Rao MR PBEF1/NAmPRTase/Visfatin: a potential malignant astrocytoma/glioblastoma serum marker with prognostic value Cancer Biol Ther 2008 7 5 663 668 10.4161/cbt.7.5.5663 18728403

[B6] Khan JA Tao X Tong L Molecular basis for the inhibition of human NMPRTase, a novel target for anticancer agents Nat Struct Mol Biol 2006 13 7 582 588 10.1038/nsmb1105 16783377

[B7] Hasmann M Schemainda I FK866, a highly specific noncompetitive inhibitor of nicotinamide phosphoribosyltransferase, represents a novel mechanism for induction of tumor cell apoptosis Cancer Res 2003 63 21 7436 7442 14612543

[B8] Ewing TJA Kuntz ID Critical evaluation of search algorithms for automated molecular docking and database screening Journal of Computational Chemistry 1997 18 9 1175 1189 10.1002/(SICI)1096-987X(19970715)18:9<1175::AID-JCC6>3.0.CO;2-O

[B9] Ruben A Maxim T Dmitry K ICM - A new method for protein modeling and design: Applications to docking and structure prediction from the distorted native conformation Journal of Computational Chemistry 1994 15 5 488 506 10.1002/jcc.540150503

[B10] Garrett MM David SG Robert SH Ruth H William EH Richard KB Arthur JO Automated docking using a Lamarckian genetic algorithm and an empirical binding free energy function Journal of Computational Chemistry 1998 19 14 1639 1662 10.1002/(SICI)1096-987X(19981115)19:14<1639::AID-JCC10>3.0.CO;2-B

[B11] Khodade P Prabhu R Chandra N Raha S Govindarajan R Parallel implementation of AutoDock Journal of Applied Crystallography 2007 40 3 598 599 10.1107/S0021889807011053

[B12] Lipinski CA Lombardo F Dominy BW Feeney PJ Experimental and computational approaches to estimate solubility and permeability in drug discovery and development settings Adv Drug Deliv Rev 2001 46 1-3 3 26 10.1016/S0169-409X(00)00129-0 11259830

[B13] LIGPREP http://www.schrodinger.com/ProductDescription.php?mID=6&amp;sID=7&cID=0

[B14] Bernstein FC Koetzle TF Williams GJ Meyer EF Jr Brice MD Rodgers JR Kennard O Shimanouchi T Tasumi M The Protein Data Bank: a computer-based archival file for macromolecular structures J Mol Biol 1977 112 3 535 542 10.1016/S0022-2836(77)80200-3 875032

[B15] Singh UC Peter AK An approach to computing electrostatic charges for molecules Journal of Computational Chemistry 1984 5 2 129 145 10.1002/jcc.540050204

[B16] Sobolev V Sorokine A Prilusky J Abola EE Edelman M Automated analysis of interatomic contacts in proteins Bioinformatics 1999 15 4 327 332 10.1093/bioinformatics/15.4.327 10320401

[B17] Brunger AT Adams PD Clore GM DeLano WL Gros P Grosse-Kunstleve RW Jiang JS Kuszewski J Nilges M Pannu NS Crystallography & NMR system: A new software suite for macromolecular structure determination Acta Crystallogr D Biol Crystallogr 1998 54 Pt 5 905 921 10.1107/S0907444998003254 9757107

[B18] Kleywegt GJZJ Kjeldgaard M Jones TA Rossmann MG AE Around O International Tables for Crystallography, Vol F Crystallography of Biological Macromolecules 2001 Dordrecht: Kluwer Academic Publishers, The Netherlands 353 356 366 367.

[B19] The PyMOL Molecular Graphics System http://www.pymol.org

[B20] Das S El-Deiry WS Somasundaram K Efficient growth inhibition of HPV 16 E6-expressing cells by an adenovirus-expressing p53 homologue p73beta Oncogene 2003 22 52 8394 8402 10.1038/sj.onc.1206908 14627980

[B21] Yeturu K Chandra N PocketMatch: a new algorithm to compare binding sites in protein structures BMC Bioinformatics 2008 9 543 10.1186/1471-2105-9-543 19091072 PMC2639437

[B22] Colombano G Travelli C Galli U Caldarelli A Chini MG Canonico PL Sorba G Bifulco G Tron GC Genazzani AA A novel potent nicotinamide phosphoribosyltransferase inhibitor synthesized via click chemistry J Med Chem 2010 53 2 616 623 10.1021/jm9010669 19961183

