# Supplementary material for: Virtual screening, identification and experimental testing of novel inhibitors of PBEF1/Visfatin/NMPRTase for glioma therapy
Source: J Clin Bioinforma. 2011 Jan 20;1:5. doi: 10.1186/2043-9113-1-5 (PMC3143896; doi:10.1186/2043-9113-1-5)
Supplement: Additional file 1 — Figure S1 Binding site of the six identified compounds and the two control compounds. Figure showing the binding sites of all the top six compounds along with the control compounds, NMN and FK866. [file 2043-9113-1-5-S1.DOC]

Supporting information:

Figure S1 - Docked poses for the potential lead compounds identified after virtual screening


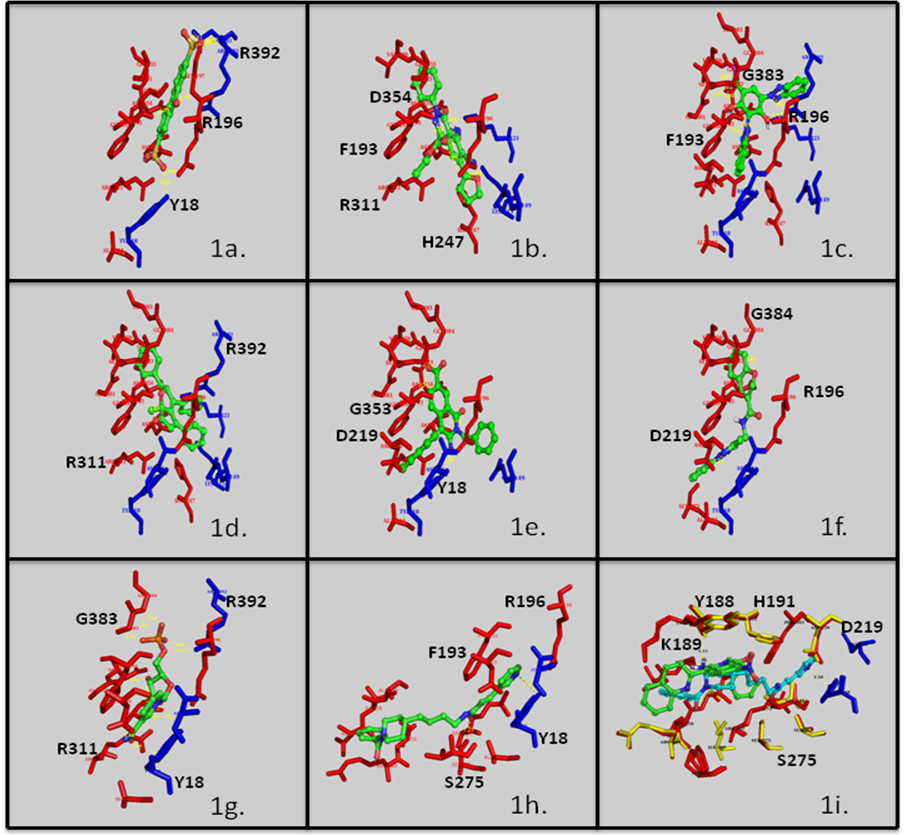


***Figure S1****: Docked poses for the potential lead compounds identified after virtual screening (Fig. 1a-1f). The first binding mode is shown for all compounds, whereas the second mode is shown only for compound 5, which was the most efficacious lead molecule among the six.* ***a****: compound 1,* ***b****: compound 2, c: compound 3, d: compound 4, e: compound 5 , f: compound 6, all in mode 1. The crystallographically observed poses of NMN and FK866 are shown in 1g and Fig 1h respectively. Fig 1i shows the superposition of the FK866 site with the second mode site of compound 5. The ligands are in ball and stick model and colored by standard atom types; the A chain residues are shown in red and C chain residues are in blue, in all the panels. The residues in the A chain common between FK866 binding and second mode binding of compound 5 are shown in yellow sticks in Fig 1i..*
